# 7 T MRI in moyamoya disease: current evidence and AI-assisted future directions—a narrative review

**DOI:** 10.3389/fneur.2026.1941574

**Published:** 2026-10-13

**Authors:** Haiyang Wu, Jingchun Wang, Zhenhua Wang, Changfa Zhao, Rui Zhang, Shuai Jiang, Qingbao Guo

**Affiliations:** Department of Neurosurgery, Xi’an No. 9 Hospital, Xi’an, Shaanxi, China

**Keywords:** 7 T MRI, artificial intelligence, moyamoya disease, risk stratification, vascular phenotyping

## Abstract

**Figure:**
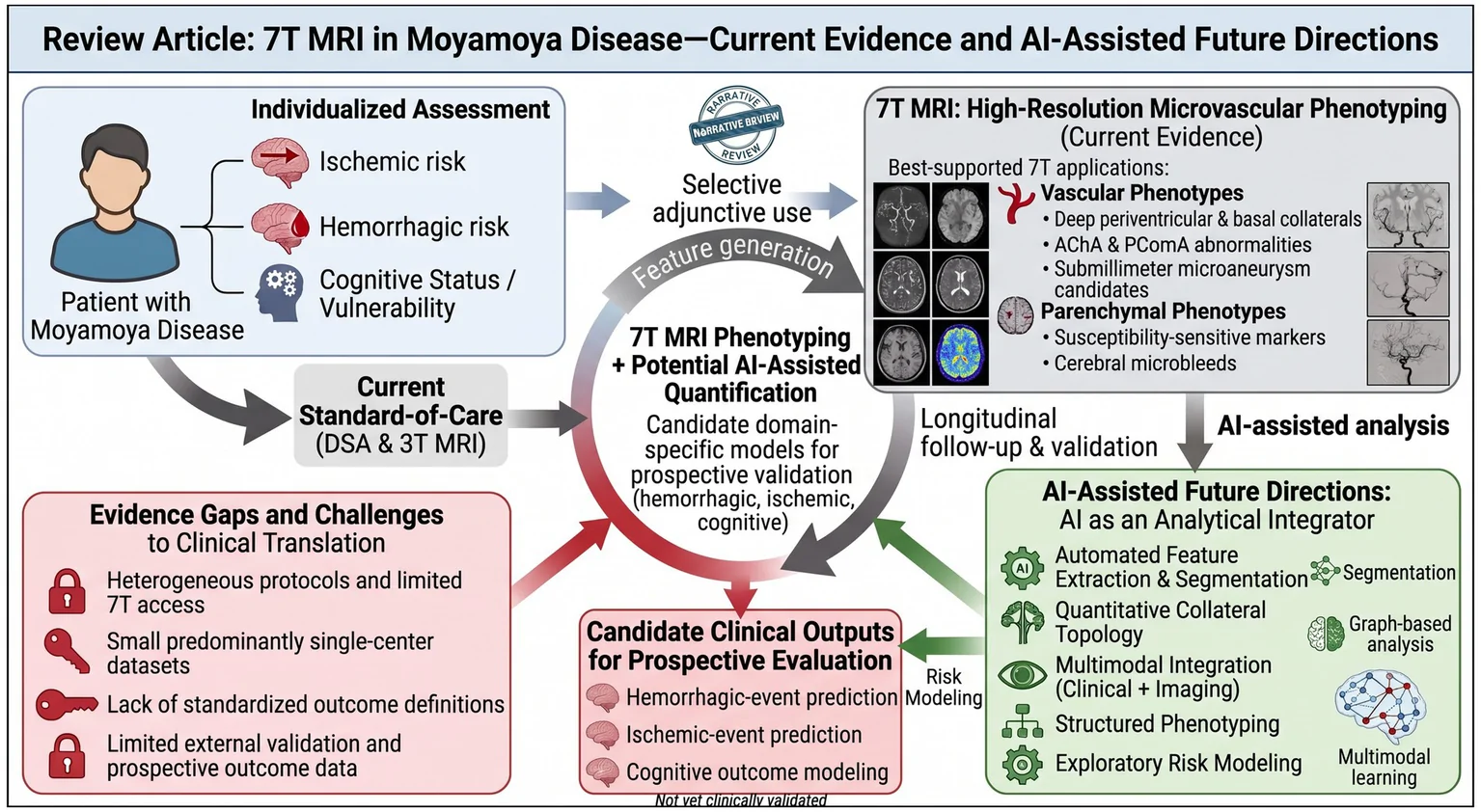

## 1 Introduction

Moyamoya disease (MMD) is an idiopathic progressive steno-occlusive arteriopathy involving the terminal intracranial internal carotid arteries and their proximal branches. Progressive arterial narrowing is accompanied by the development of fragile basal and periventricular collateral networks that produce the characteristic angiographic appearance described as a “puff of smoke” (1–3). Clinical presentation varies by age and vascular phenotype and includes transient ischemic attacks, ischemic stroke, intracranial hemorrhage, headache, seizures, and cognitive impairment. Although the pathogenesis remains incompletely understood, genetic susceptibility, including RNF213-related mechanisms, and dysregulated vascular remodeling are increasingly recognized (4).

A central challenge in MMD is prospective risk stratification. Ischemic events reflect a complex interaction among arterial stenosis, impaired cerebrovascular reserve, collateral compensation, and embolic or watershed mechanisms. Hemorrhagic events are more closely linked to fragile deep collateral remodeling, periventricular anastomoses, choroidal or posterior communicating artery enlargement, microaneurysm formation, and prior microbleeding. Cognitive decline may develop even without overt stroke through chronic hypoperfusion, repeated subclinical injury, white-matter disruption, and network dysfunction (3, 5). These ischemic, hemorrhagic, and cognitive domains overlap but are not interchangeable; a single angiographic stage or qualitative imaging label cannot fully describe patient-level risk.

DSA remains the reference examination for defining arterial stenosis and collateral pathways, while conventional MRI and MRA are indispensable for noninvasive diagnosis, parenchymal assessment, and follow-up (3, 6). Each modality has limitations. DSA is invasive and is not ideal for repeated longitudinal phenotyping. Optimized 3 T MRI identifies major stenoses, infarction, broad collateral patterns, and many hemorrhagic markers, but very small perforating vessels, deep periventricular channels, microaneurysm candidates, subtle susceptibility lesions, and fine vessel-wall abnormalities may remain near its resolution limits. Ultimately, the translational value of higher-resolution imaging depends on its ability to provide reproducible, clinically actionable information that conventional examinations miss.

Seven-tesla MRI offers greater signal-to-noise ratio, stronger susceptibility sensitivity, and submillimeter vascular and structural imaging, creating an opportunity to characterize MMD at a scale closer to its deep collateral and microinjury pathology (7–9). Yet greater image detail also increases analytic complexity. Numerous candidate biomarkers may be visible, but manual evaluation is time-consuming, reader-dependent, and difficult to integrate across vascular, perfusion, structural, and clinical domains. AI methods may help standardize segmentation and measurement, combine heterogeneous data, and generate individualized estimates. Their value, however, depends on appropriate clinical endpoints, robust validation, calibration, interpretability, and demonstration that predictions improve management rather than merely separate retrospective groups.

This review examines the evidence for 7 T-derived vascular and parenchymal phenotypes in MMD and considers how AI could support their measurement and integration. We focus on the relative maturity of hemorrhagic, ischemic, and cognitive applications, the position of 7 T within current imaging pathways, and the evidence needed for clinically useful prediction.

Clinical value must be judged against established management pathways. Contemporary Japanese guidelines and the American Heart Association/American Stroke Association scientific statement continue to base diagnosis and treatment on clinical presentation, angiographic anatomy, conventional MRI/MRA, and hemodynamic assessment rather than on any single emerging imaging biomarker (10, 11). In hemorrhagic MMD, the Japan Adult Moyamoya Trial provided trial-level evidence that direct bypass can reduce rebleeding in selected adults, although the effect estimate was imprecise and the results were achieved within specialized surgical practice (12). This context defines the translational standard for 7 T MRI and AI: a new biomarker gains clinical relevance primarily when it alters patient management, for instance, by adjusting surveillance intensity or guiding the timing of revascularization.

## 2 Methods

This narrative review was based on a targeted search of PubMed/MEDLINE, Embase, and Web of Science for studies related to moyamoya disease, 7 T MRI, ultra-high-field MRI, artificial intelligence, machine learning, deep learning, radiomics, vascular phenotyping, and risk stratification. Original human studies, clinically relevant methodological reports, consensus or review articles, and studies addressing diagnosis, collateral characterization, hemorrhagic or ischemic risk, cognition, and translational barriers were prioritized. Because the literature is heterogeneous in imaging protocols, model architectures, validation strategies, and clinical endpoints, no formal meta-analysis or risk-of-bias assessment was performed. Evidence was synthesized according to technical maturity, disease specificity, clinical relevance, and translational readiness.

Where available, study-level quantitative results were extracted and reported, including cohort size, imaging protocol and spatial resolution, agreement with reference imaging, and diagnostic or classification performance metrics such as accuracy, sensitivity, specificity, and area under the receiver operating characteristic curve. We also distinguished direct MMD-specific evidence from technical evidence derived from other cerebrovascular populations and separated published empirical findings from forward-looking interpretation.

## 3 7 T MRI in moyamoya disease

### 3.1 Technical foundations and clinical scope

The main physical advantage of 7 T MRI is increased signal-to-noise ratio, which can be exchanged for higher spatial resolution, shorter acquisition, or improved contrast in selected sequences (7, 8). In time-of-flight MRA (TOF-MRA), prolonged blood T1 and stronger flow-related enhancement improve small-vessel conspicuity. Studies of intracranial vascular imaging have shown sharper arterial boundaries and better depiction of perforating and distal vessels at 7 T than at lower field strengths (13–18). High-resolution structural imaging also improves visualization of small neuroanatomic structures and microstructural abnormalities, although the gain is sequence- and region-dependent (9, 19–21).

These advantages are particularly relevant to MMD because the disease is defined not only by proximal stenosis but also by the morphology and spatial organization of small collateral channels. Deep lenticulostriate, thalamic, choroidal, and periventricular pathways can be difficult to separate on conventional MRA. MMD-specific work has demonstrated that 7 T imaging can depict and classify deeply seated collateral networks with greater detail (22, 23). Direct MMD-specific evidence for quantitative lenticulostriate artery imaging remains limited. Li et al. demonstrated that 7 T TOF-MRA can quantify LSA stems, branches, length, and tortuosity in 161 patients with MCA stenosis (24), supporting technical feasibility for perforator imaging; however, these findings should not be interpreted as direct validation of LSA biomarkers in MMD. Table 1 summarizes representative MMD-specific 7 T MRI studies and their quantitative findings, whereas Table 2 summarizes the potential advantages and limitations of 7 T MRI compared with 3 T MRI across clinically relevant MMD applications. Importantly, improved small-vessel conspicuity at 7 T is protocol-dependent and should not be attributed to field strength alone. In a non-MMD TOF-MRA optimization study, the contrast-to-noise ratio increased from 2.7 ± 0.9 at 3 T to 4.5 ± 0.8 at 7 T, while the mean intracranial vessel-quality score increased from 2.28 to 2.89 (13). This additional signal can be exchanged for smaller voxel dimensions; for example, MMD-specific studies have used isotropic 0.3-mm TOF-MRA (22) or 0.22 × 0.22 × 0.41 mm^3^ acquisitions (23, 25). However, voxel size, slab orientation and coverage, flip angle, radiofrequency transmit efficiency, parallel imaging or reconstruction strategy, motion, and slow or complex flow can all alter TOF signal and apparent vessel continuity. Consequently, measurements of vessel number, caliber, or branching should not be pooled across centers without acquisition and reconstruction harmonization.

**Table 1:** Representative MMD-specific 7 T MRI studies and quantitative findings.

Study	Cohort/design	7 T technique	Comparator	Main quantitative findings	Main limitation
Duan et al., 2025 (22)	180 patients, 355 hemispheres; paired 7 T/3 T; DSA subset	7 T TOF-MRA, 0.3-mm isotropic	3 T TOF-MRA and DSA	AChA visible in 45.7% vs. 3.3% at 3 T; PComA 32.4% vs. 6.2%; 42.4% of AChA and 27.7% of PComA abnormalities seen at 7 T were absent at 3 T. Agreement with DSA: *κ* = 0.891 for AChA and 0.761 for PComA	Cross-sectional; association with hemorrhagic phenotype rather than prospective prediction
Matsushige et al., 2018 (23)	15 patients, 32 affected hemispheres	7 T TOF-MRA, 0.22 × 0.22 × 0.41 mm^3^	DSA	106 deeply seated collateral networks on 7 T TOF-MRA vs. 100 on DSA; 96/100 DSA-defined networks reproduced on 7 T; six collateral pathways characterized	Small single-center cohort
Matsushige et al., 2016 (25)	10 patients	7 T TOF-MRA, 0.22 × 0.22 × 0.41 mm^3^	Conventional MRA/DSA	Four ventricular microaneurysms detected; diameter 0.56–0.96 mm; not detected by conventional MRA or DSA	Very small feasibility cohort; uncertain natural history

MMD, moyamoya disease; TOF-MRA, time-of-flight magnetic resonance angiography; DSA, digital subtraction angiography; AChA, anterior choroidal artery; PComA, posterior communicating artery; MPRAGE, magnetization-prepared rapid gradient echo.

**Table 2:** Potential advantages and limitations of 7 T MRI compared with 3 T MRI in moyamoya disease.

Dimension	3 T	7 T	Value for MMD evaluation
Fundamental Physical Advantages	Provides adequate clinical diagnostic capability in most scenarios.	Higher SNR/CNR enabling ultra-high spatial resolution; literature demonstrates SNR gain (~2.3 × compared to 3 T) (8, 26).	Enables visualization of tiny collateral channels, distal arteries, microhemorrhage, and microinfarcts not well detected at 3 T (8, 21, 26).
TOF-MRA (Non-contrast MRA)	Major branches visualized; limited visualization of tiny collaterals and distal networks.	Increased SNR and prolonged blood T1 at 7 T enhance flow-related signal and small-vessel contrast (16, 17).	In MMD cohort studies: greater number of collateral flow voids on T2 (3.71 vs. 1.17) and higher TOF collateral signal (7.16 vs. 4.75) vs. 3 T; achievable resolution 0.22 × 0.22 × 0.41 mm^3^ for deep collaterals (17).
Deep/Perforating Collaterals	Visualization of lenticulostriate and choroidal collaterals remains limited.	Improved depiction of deep collateral networks and perforators (17, 21).	May support anatomical characterization relevant to risk stratification and surgical planning; prospective clinical utility remains unproven (17, 21).
SWI/Susceptibility-Based Imaging	Detects microbleeds and venous structures, but sensitivity to minute lesions is limited.	Stronger susceptibility effect and higher SNR optimize microvascular lesion depiction (8, 16, 17, 21).	For hemorrhagic MMD, 7 T SWI + TOF improves detection of tiny collateral aneurysms (0.56–0.96 mm) and identification of bleeding-responsible vessels (17).
Vessel Wall Imaging	Can differentiate atherosclerosis vs. inflammatory disease, but distal small-caliber wall visualization limited.	Better depiction of thin vascular walls and distal segments due to high spatial resolution (16, 18).	Assists in differentiating MMD vs. atherosclerosis vs. vasculitis, especially in atypical phenotypes (16, 18).
Parenchymal micro-injury	Detects most cortical/subcortical infarcts, but microinfarct detection is limited.	Submillimeter resolution allows improved detection of cortical microinfarcts and micro-ischemic burden (8, 21, 26).	May improve quantification of microstructural injury; its incremental prognostic value over optimized 3 T MRI remains unproven (8, 21, 26).
Perfusion/Hemodynamics (ASL, PWI, 4D flow)	Mature and widely implemented in clinical follow-up.	Technically feasible but faces workflow limitations, SAR constraints, and standardization challenges (16, 17, 26, 27).	May provide higher-resolution characterization of collateral compensation and post-bypass hemodynamics, although MMD-specific clinical validation remains limited (16, 17, 26, 27).
Technical Limitations and Accessibility	Widely accessible; standardized protocols, robust clinical evidence	radiofrequency transmit-field (B1+) inhomogeneity, SAR limitation, stronger susceptibility artifacts; requires high expertise (8, 16, 27).	7 T is best used as an adjunct high-precision evaluation (bleeding source, deep collaterals) rather than routine first-line imaging (8, 17, 27).

MMD, moyamoya disease; SNR, signal-to-noise ratio; CNR, contrast-to-noise ratio; TOF-MRA, time-of-flight magnetic resonance angiography; SWI, susceptibility-weighted imaging; SAR, specific absorption rate.

The technical gain is accompanied by important constraints. Radiofrequency transmit-field (B1+) inhomogeneity, higher specific absorption rate, susceptibility-related distortion, motion sensitivity, and signal loss in very slow or complex flow can degrade image quality (14, 16–18, 26, 27). Acquisition and reconstruction remain less standardized than at 3 T, and access to 7 T systems and experienced personnel is limited. These limitations argue against viewing 7 T as a universal first-line examination. Its near-term role is more plausibly an adjunctive, high-precision tool for questions that remain unresolved after optimized 3 T MRI and DSA.

### 3.2 Deep collateral remodeling and hemorrhagic phenotyping

The strongest quantitative MMD-specific evidence comes from Duan et al. (22). In a cross-sectional cohort of 180 patients comprising 355 hemispheres, 7 T TOF-MRA visualized the anterior choroidal artery (AChA) in 45.7% of hemispheres and the posterior communicating artery (PComA) in 32.4%, compared with only 3.3 and 6.2%, respectively, on routine 3 T TOF-MRA. Accordingly, 42.4% of AChA abnormalities and 27.7% of PComA abnormalities identified at 7 T were not visualized at 3 T. Among 37 patients (70 hemispheres) who also underwent DSA, 7 T TOF-MRA showed strong agreement with DSA for AChA and PComA staging (weighted *κ* = 0.891 and 0.761, respectively), whereas agreement between 3 T TOF-MRA and DSA was substantially lower (weighted *κ* = 0.262 and 0.221). Higher AChA and PComA grades were associated with hemorrhagic presentation, with adjusted odds ratios of 1.90 and 3.89, respectively. Because the study was cross-sectional, these findings should be interpreted as associations with hemorrhagic phenotype rather than validated prospective predictors of future hemorrhage.

Matsushige et al. (23) provided complementary morphological evidence in 15 patients with 32 affected hemispheres. Using 7 T TOF-MRA with a spatial resolution of 0.22 × 0.22 × 0.41 mm^3^, the investigators identified 106 deeply seated collateral networks, compared with 100 on DSA and 73 on 7 T MPRAGE. Ninety-six of the 100 collateral networks identified on DSA were reproduced on 7 T TOF-MRA. Six collateral pathways were characterized, including striatal or choroidal connections to cortical arteries, transcallosal pathways, choroidal/PComA-to-thalamostriate anastomoses, intrastriatal and intrathalamic connections, and basal cisternal moyamoya networks. These data support improved morphological characterization at 7 T, although the small, single-center cohort limits generalizability.

In a separate feasibility study of 10 patients, Matsushige et al. (25) identified four ventricular microaneurysms arising from collateral vessels using 7 T TOF-MRA at 0.22 × 0.22 × 0.41 mm^3^ resolution. The microaneurysms measured 0.56–0.96 mm in diameter and were not identified on conventional MRA or DSA. One lesion disappeared 6 months after direct bypass surgery. Although these observations demonstrate the ability of 7 T MRI to depict submillimeter vascular abnormalities, they do not establish the prevalence, natural history, or treatment implications of such lesions.

Susceptibility-sensitive imaging provides a complementary parenchymal marker of vascular fragility. Higher field strength increases sensitivity to hemosiderin and small venous structures, and comparative studies have shown increased detection of cerebral microbleeds at 7 T (28, 29). Experience in other hemorrhage-prone small-vessel disorders also illustrates the ability of 7 T MRI to reveal subtle vascular and microhemorrhagic markers. In MMD, combining microbleed burden, collateral topology, and microaneurysm candidates may be more informative than evaluating any single marker (30). Prospective studies must determine whether this combination improves hemorrhage prediction beyond prior hemorrhage, DSA collateral patterns, and optimized 3 T susceptibility imaging.

Collectively, these studies demonstrate that 7 T MRI can increase the conspicuity of deep collateral pathways, AChA/PComA abnormalities, and submillimeter vascular outpouchings. However, improved visualization should not be equated with improved prognosis or treatment selection. Greater sensitivity may reveal lesions of uncertain biological significance, whereas very slow or complex flow can still produce signal loss and false-negative appearances. The available studies are predominantly small or single-center, use heterogeneous acquisition protocols, and rarely link 7 T findings to prespecified longitudinal outcomes. Thus, the current evidence supports improved vascular phenotyping, but not yet outcome-based clinical superiority over optimized 3 T MRI and DSA.

### 3.3 Hemodynamic, vessel-wall, and parenchymal phenotyping

For ischemic risk, the relevant target is not stenosis alone but the adequacy of compensatory flow and cerebrovascular reserve. Seven-tesla MRI may improve spatial localization of distal arterial attenuation, slow-flow territories, and microstructural consequences of chronic hypoperfusion. High-field approaches to cerebrovascular reactivity and perfusion can reveal regional abnormalities at finer spatial scales, but susceptibility to transit delay, field inhomogeneity, and protocol variation remains substantial (26, 31). Arterial spin labeling, resting-state functional MRI, blood oxygen level-dependent methods, and 4D-flow techniques at 7 T remain best considered research tools until acquisition and analysis are standardized and validated against established hemodynamic reference methods.

Intracranial vessel-wall imaging is another emerging application. At 3 T, high-resolution wall MRI can help distinguish MMD from intracranial atherosclerosis by differences in remodeling, wall thickening, and enhancement (32–34). The higher spatial resolution of 7 T may improve visualization of thin distal arterial walls, but dedicated MMD evidence remains limited. A 7 T vessel-wall radiomics study in intracranial atherosclerosis demonstrates technical feasibility rather than direct clinical validity in MMD (35). Claims regarding disease activity, inflammatory change, or fibrocellular remodeling at 7 T should therefore remain hypothesis-generating.

Parenchymal imaging may capture cumulative injury that is not visible as a territorial infarct. Ultra-high-field structural and susceptibility imaging can improve detection of microinfarcts, small hemorrhagic residues, perivascular spaces, cortical changes, and white-matter abnormalities (19–21, 26, 29). These findings may be relevant to cognitive dysfunction, but their incremental value over optimized 3 T imaging and neuropsychological assessment is unknown. The most defensible interpretation is that 7 T MRI expands the range of candidate MMD phenotypes; it has not yet established which phenotypes are sufficiently reproducible and prognostic to guide routine care.

### 3.4 Positioning 7 T within current clinical imaging pathways

Current MMD imaging is multimodal. DSA provides detailed assessment of luminal stenosis, transdural supply, and collateral architecture, whereas MRI/MRA supports parenchymal evaluation and noninvasive follow-up. Current Japanese and American guidance does not assign a routine role to 7 T MRI, reflecting its limited availability and the absence of outcome-based evidence (10, 11). A distinction is also required between intracranial deep collateral pathways and extracranial-to-intracranial transdural or meningeal collateralization. Current 7 T MMD studies have primarily characterized intracranial pathways, including choroidal, PComA, striatal, thalamic, and periventricular networks. Although high-resolution intracranial TOF-MRA may depict distal recipient vessels or intracranial portions of transdural pathways, it does not provide a comprehensive assessment of the entire ECA-derived collateral circulation. Limited acquisition coverage, flow-direction dependence, slow-flow signal loss, and the absence of selective temporal information remain important constraints. Therefore, DSA remains the reference technique when comprehensive mapping of ECA-derived transdural or meningeal supply is required, particularly for surgical planning.

In the near term, 7 T MRI is most likely to serve as a selective adjunct when optimized 3 T MRI leaves uncertainty regarding deep collateral anatomy, microaneurysmal pathology, or microbleed burden. Very slow or complex flow may still produce signal loss on TOF-MRA, and findings of uncertain natural history require confirmation against DSA or follow-up imaging.

## 4 Artificial intelligence in moyamoya disease

### 4.1 Diagnosis and structured imaging phenotyping

Most AI studies in MMD have focused on diagnosis. Convolutional neural networks have detected MMD-related patterns on skull radiographs, T2-weighted MRI, MRA, and DSA with high reported discrimination (36–41). Retinal photography and cerebral oxygen saturation signals have also been explored as nontraditional screening inputs (42, 43). Table 3 summarizes representative diagnostic studies. Recent multicenter work using T2-weighted MRI included independent external validation across several hospitals and illustrates progress beyond single-center proof-of-concept development (44). Even so, the primary task remained screening for moyamoya angiopathy rather than predicting future events or treatment response. These results demonstrate that disease-related imaging patterns are learnable, but they do not establish that AI improves diagnosis in patients already undergoing specialist neurovascular imaging.

**Table 3:** Representative AI-driven diagnostic studies in moyamoya disease.

Study	Modality and cohort	AI method	Key result	Validation design
Kim, 2019 (36)	Skull X-ray (345 MMD, 408 controls)	CNN (ResNet-based)	Internal: accuracy 84.1%, sensitivity 84%, specificity 84%, AUROC 0.91; independent external accuracy 75.9%	Held-out institutional test set (30%) plus independent external validation at a geographically separate hospital
Lei, 2021 (38)	DSA (single-center retrospective cohort)	ResNet-152	Diagnosis: accuracy 97.64%, sensitivity 96.55%, specificity 98.29%, AUC 0.990; hemorrhagic-phenotype classification: accuracy 90.69%, sensitivity 94.12%, specificity 89.86%	Five-fold cross-validation for diagnostic classification; repeated patient-level 80:20 train/validation splits for hemorrhagic-phenotype classification; no independent external validation
Akiyama, 2020 (40)	Brain MRI (T2WI)	VGG16	Accuracy 92.8, 84.8, and 87.8% at basal cistern, basal ganglia, and centrum semiovale levels	Single-center random learning/validation split (10–20% validation depending on group); no independent external validation
Lu, 2024 (41)	TOF-MRA	DenseNet-121	Internal macro-AUC 0.977; external macro-AUC 0.880; MMD-targeted binary classification accuracy 0.967 in the internal test set	Internal train/validation/test split (75:15:10) plus independent external validation at a separate institution
Hong, 2024 (42)	Fundus photographs	ResNeXt-50	AUC 0.946; sensitivity 89.8%; specificity 90.4%	Single-center held-out patient-level test set (80:20) with repeated age-matched training/validation sampling; no independent external validation

These studies vary in cohort size, validation design, imaging modality, and clinical endpoint; therefore, reported performance metrics should not be directly compared across studies.

MMD, moyamoya disease; CNN, convolutional neural networks; AUC, area under curve; DSA, digital subtraction angiography; TOF-MRA, time-of-flight magnetic resonance angiography; AUC/AUROC, area under the receiver operating characteristic curve.

The more important translational opportunity is structured phenotyping. Moving beyond simple diagnostic labels, AI algorithms could be tailored to extract structured, actionable biomarkers. Examples include mapping deep collateral pathways, quantifying microbleed burden, or profiling vessel-wall morphology. These outputs are closer to the features used in clinical reasoning and can be linked to specific outcomes. They also permit human review and error analysis, which are difficult when a model provides only a global class probability.

Differential diagnosis remains a clinically relevant task, particularly in adults with coexisting vascular risk factors. Models should distinguish idiopathic MMD from intracranial atherosclerosis, vasculitis, and secondary moyamoya-like vasculopathies rather than from healthy controls alone. Training data must therefore represent the actual diagnostic spectrum encountered in practice. Performance in highly curated case–control datasets may not generalize to atypical or mixed phenotypes.

### 4.2 Domain-specific phenotyping and exploratory risk modeling

The clinical value of AI will depend on prediction of outcomes that influence management. For hemorrhagic risk, multimodal models have combined clinical variables and angiographic features to estimate hemisphere-specific risk, while image-based models have identified deep white-matter and periventricular collateral regions associated with hemorrhagic presentation (38, 45). Machine-learning analyses have also examined factors associated with periventricular aneurysm formation, hemorrhagic phenotype, and stroke recurrence (46, 47). Table 4 summarizes representative AI/ML studies spanning disease phenotyping, cognitive classification, and exploratory risk stratification. A 2026 systematic review and meta-analysis of seven retrospective cohorts reported moderate-to-high aggregate discrimination for AI-based stroke-event estimation but also found pooled sensitivity of only 0.65, underscoring the risk of missed high-risk patients and the early stage of the evidence (48). The convergence of model attention on periventricular and choroidal pathways is biologically plausible, but most primary studies predict retrospective phenotype or composite stroke status rather than a prespecified future event.

**Table 4:** Representative AI/ML studies for disease phenotyping, cognitive classification, and exploratory risk stratification in moyamoya disease.

Risk domain/endpoint	Study	Input data	Model	Key findings	Clinical take-home message
Retrospective hemorrhagic-phenotype classification	Lei et al., 2021 (38)	Clinical variables (age, sex, hypertension, smoking) + DSA features (aneurysm, collaterals)	Multi-view CNN (MV-CNN-C)	Hemorrhagic-phenotype classification: accuracy 90.69%, sensitivity 94.12%, and specificity 89.86%	Demonstrates the feasibility of hemisphere-level multimodal hemorrhagic-phenotype classification; prospective hemorrhage prediction and intervention utility remain unproven
Hemorrhagic vs. ischemic phenotype classification	Yin et al., 2022 (45)	MRA collateral appearance across the basal cistern, basal ganglia, and centrum semiovale	Transfer-learning ResNet-18	>91% accuracy at basal cistern/basal ganglia levels; Grad-CAM highlights deep white matter/periventricular collateral regions	Model attention concentrated on deep/periventricular regions, supporting their relevance to hemorrhagic phenotyping; causal or prospective prognostic significance remains unproven.
Hemorrhagic risk factors + stroke recurrence	Chen et al., 2021 (47)	Clinical-demographic + angiographic factors	XGBoost + other ML (LR/SVM/CatBoost)	Hemorrhage predictors: advanced Suzuki stage, intracranial aneurysm, rural residence, frequent hospitalization, younger onset; recurrence linked to advanced stage, age 18–44, no prior revascularization, aneurysm	Identifies nonlinear associations among clinical, demographic, and angiographic variables; prospective recurrence prediction and external validity remain to be established
Structural disease phenotyping	Tompkins et al., 2021 (49)	Structural MRI cortical thickness features	SVM/Random forest	Cortical-thickness abnormalities were identified in multiple regions; machine-learning models distinguished MMD from controls, with mean overall accuracy of approximately 0.65–0.79 depending on the algorithm.	Supports structural MRI phenotyping of MMD, but the study did not include neuropsychological labels or longitudinal cognitive outcomes and therefore should not be interpreted as a cognitive-risk prediction model.
Cognitive impairment classification (functional)	Lei et al., 2020 (50)	Resting-state fMRI dynamic functional connectivity	ML classifiers	VCI vs. non-VCI: AUC 0.9102, accuracy 84.81%, sensitivity 80.56%, specificity 88.37%	Demonstrates the feasibility of rs-fMRI-based VCI classification; longitudinal prediction of cognitive decline remains unproven
Cognitive impairment classification (metabolic)	Weng et al., 2023 (51)	18F-FDG PET	Sparse-representation classifier	Accuracy 82.4%; selected-feature model AUC 0.879, sensitivity 67.9%, specificity 91.2%	Supports PET-based cross-sectional cognitive classification; longitudinal prognostic utility remains unproven

Most models remain exploratory and require external validation, calibration, and prospective assessment before clinical deployment.

MMD, moyamoya disease; MRA, magnetic resonance angiography; MV-CNN-C, multi-view convolutional neural networks; ML, machine learning; SVM, support vector machine.

For ischemic risk, clinically meaningful outputs include recurrent ischemic events, impaired cerebrovascular reserve, progressive arterial compromise, and likely benefit from revascularization. Models that classify symptomatic status or angiographic stage are less useful unless they add information beyond conventional interpretation. Future studies should compare AI predictions against baseline clinical-imaging models and test whether the model changes surveillance intensity, selection for hemodynamic testing, or timing of surgery.

Current AI-related cognitive studies in MMD do not yet constitute longitudinal cognitive-risk prediction. Tompkins et al. analyzed structural T1 MRI from 269 MMD examinations and 993 control examinations and demonstrated cortical-thickness abnormalities with machine-learning discrimination between MMD and controls (49). However, neuropsychological outcomes were not available, and this work is more appropriately considered structural disease phenotyping rather than cognitive-risk modeling.

Subsequent studies linked imaging features more directly to neuropsychologically defined cognitive impairment. Lei et al. included 36 patients with vascular cognitive impairment (VCI), 43 patients with intact cognition, and 26 normal controls, and used dynamic resting-state functional connectivity with a sparse-representation classifier (50). For VCI versus non-VCI classification, the model achieved an AUC of 0.9102, accuracy of 84.81%, sensitivity of 80.56%, and specificity of 88.37%. Weng et al. classified 152 adults with MMD into VCI (*n* = 100) and non-VCI (*n* = 52) groups according to multidomain neuropsychological testing and combined these labels with 18F-FDG PET (51). Their sparse-representation classifier achieved an accuracy of 82.4%; a selected-feature model yielded an AUC of 0.879, sensitivity of 67.9%, and specificity of 91.2%.

These studies suggest an evolution from structural disease phenotyping toward functional and metabolic classification anchored to neuropsychological assessment. Nevertheless, they remain classification studies rather than prospective models of future cognitive decline. Longitudinal studies should therefore use standardized domain-specific neuropsychological outcomes and explicitly account for education, prior stroke, mood, treatment, and postoperative change.

### 4.3 Imaging-omics and biomarker discovery

AI-assisted molecular studies may provide biological context for imaging phenotypes, although they are currently remote from clinical deployment. Machine-learning analysis of serum metabolomic profiles has identified candidate diagnostic signatures, and transcriptomic studies have prioritized genes related to extracellular matrix organization, mitochondrial function, immune signaling, and vascular remodeling (52–54). These studies remain vulnerable to batch effects, population-specific confounding, and unstable feature selection. Their immediate value is hypothesis generation: future imaging-omics studies could test whether 7 T-derived collateral morphology, microbleed burden, vessel-wall features, or perfusion-reserve deficits correspond to molecular signatures of vascular fragility or ischemic stress.

### 4.4 Methodological and reporting problems

The apparently high accuracy of current AI studies must be interpreted within their design. Many are retrospective, single-center, or case–control studies with small samples, curated images, and internal validation. Scanner vendor, sequence parameters, disease severity, referral pattern, and treatment status may become hidden predictors. Class imbalance and repeated examinations from the same patient can further inflate performance if splitting occurs at the image rather than patient level. The recent multicenter T2-weighted imaging study shows that geographically separate validation is feasible, but its lower accuracy in milder angiographic disease also demonstrates spectrum dependence (44).

Because patients may contribute bilateral hemispheres and repeated examinations, dataset splitting must occur at the patient level. External validation should include different institutions, scanners, disease stages, and clinically relevant alternative arteriopathies. CLAIM and TRIPOD+AI provide appropriate reporting frameworks for these studies (55, 56).

Performance reporting should move beyond accuracy and area under the receiver operating characteristic curve. Calibration is essential when predicted probabilities are used for counseling, surveillance, or treatment decisions. Performance reporting should include positive and negative predictive values at clinically relevant thresholds. Decision-curve analysis can estimate whether a model provides net benefit over current practice, but it cannot compensate for biased sampling or poorly defined outcomes. In a rare disease, a model developed in an enriched tertiary-center cohort may have a high AUC yet a low positive predictive value in screening or routine follow-up populations.

Interpretability must be matched to intended use. Saliency maps may indicate image regions associated with a decision but do not establish causal relevance. Structured outputs, such as segmented collaterals, vessel counts, microbleed maps, or perfusion territories, are more directly auditable and can be compared with expert measurements. Reproducibility also requires transparent reporting of data exclusion, label generation, feature selection, software versions, model locking, and compute requirements. Uncertainty estimates and explicit identification of out-of-distribution cases are preferable to forcing confident predictions in unfamiliar data.

Clinical evaluation requires a separate stage of evidence. DECIDE-AI recommends early live evaluation of AI decision-support systems with attention to workflow, human-computer interaction, safety, and unintended consequences (57). For MMD, a prospective study should document who sees the model output, when it is shown, whether it changes the interpretation or treatment plan, and how disagreements between clinicians and the model are resolved. A technically accurate model can still fail if it increases reading time, generates excessive false alarms, or performs differently across age, ancestry, disease stage, and imaging platform.

## 5 AI-assisted future directions for 7 T MRI in moyamoya disease

The following section is intentionally forward-looking. No published study has yet validated a clinical decision-support model that directly integrates 7 T-derived MMD biomarkers with AI and longitudinal patient outcomes.

### 5.1 Hemorrhagic-risk integration

A disease-specific integration strategy should begin with the biological pathway of interest. For hemorrhagic MMD, 7 T-derived inputs could include AChA/PComA enlargement, periventricular anastomotic pathways, collateral branching complexity, microaneurysm candidates, and microbleed number and location (22, 23, 25, 28, 29). AI could support vessel segmentation, graph-based collateral analysis, lesion detection, and spatial integration of vascular and parenchymal markers. Clinically, a model yields the most value when it provides a calibrated probability of future hemorrhagic events, rather than a basic binary classification of current scans.

Such a model must be compared with simpler predictors, including prior hemorrhage, aneurysm presence, DSA findings, and conventional MRI. An integrated model is useful only if it improves prediction or decision-making beyond these established variables. False-positive microaneurysm detection could lead to unnecessary angiography or intervention, whereas false-negative classification could provide inappropriate reassurance. Operating thresholds should therefore be evaluated according to the clinical consequences of error.

### 5.2 Ischemic-risk integration

For ischemic risk, potential inputs include distal arterial caliber, collateral compensation, perfusion-reserve surrogates, white-matter injury, and clinical presentation. AI may help combine high-resolution vascular morphology with perfusion and clinical variables to identify territories at risk of recurrent ischemia or to estimate likely benefit from revascularization. Delayed arterial transit, blood pressure, collateral adaptation, and revascularization all modify subsequent ischemic risk. Models should therefore define a baseline examination, predict prespecified longitudinal outcomes, and account for treatment during follow-up. Evaluation should include temporal and geographic external validation and comparison with angiographic staging, cerebrovascular reserve testing, and clinical judgment. Postoperative applications, including assessment of bypass-related flow redistribution, remain exploratory.

### 5.3 Cognitive and chronic-injury integration

For cognitive outcomes, 7 T MRI may contribute high-resolution markers of microinfarction, cortical and white-matter injury, microbleeds, and vascular topology. AI could integrate these features with functional connectivity, metabolic imaging, hemodynamic measures, and neuropsychological data (49–51). The main challenge is endpoint definition. Cognitive impairment in MMD is multidimensional and can be influenced by prior stroke, education, psychiatric symptoms, medications, and postoperative change. Models should therefore predict validated domain-specific cognitive outcomes rather than a loosely defined binary label.

### 5.4 Proposed workflow

Figure 1 summarizes a proposed research workflow linking standardized 7 T image acquisition, disease-specific feature extraction, multimodal modeling, and domain-specific outcome prediction. It is intended as a framework for future validation rather than a current clinical pathway.

**Figure 1:**
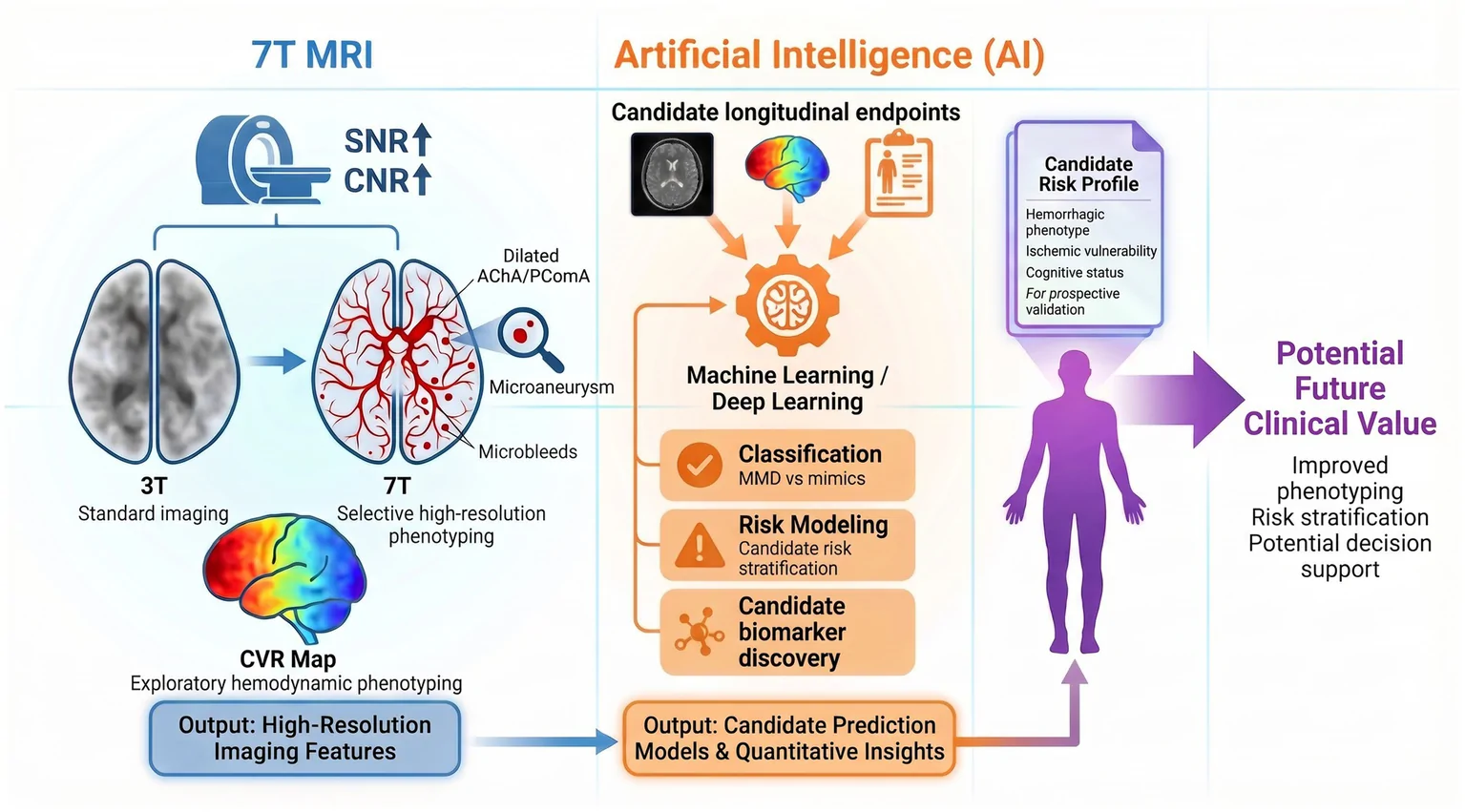
Conceptual workflow for the potential integration of 7T MRI and artificial intelligence (AI) in moyamoya disease. The left panel illustrates the technical advantages of 7T MRI (increased signal-to-noise [SNR] and contrast-to-noise ratios [CNR]) over standard 3T imaging. Acting as a selective high-resolution phenotyping tool, 7T MRI improves the visualization of dilated anterior choroidal/posterior communicating arteries (AChA/PComA), microaneurysms, and microbleeds, alongside exploratory hemodynamic phenotyping (e.g., CVR maps). The middle panel outlines the potential of AI (machine learning/deep learning) to process candidate longitudinal endpoints, driving classification tasks (MMD vs. mimics), risk modeling, and candidate biomarker discovery. The right panel projects the translational goal: generating a candidate risk profile encompassing hemorrhagic phenotype, ischemic vulnerability, and cognitive status to achieve potential future clinical value through improved phenotyping, risk stratification, and decision support. Importantly, this integrated workflow remains a conceptual framework that requires rigorous prospective longitudinal validation.

## 6 Discussion

### 6.1 State of the art: established components and conceptual integration

Evidence in MMD is currently strongest for 7 T visualization of deep collateral pathways, ventricular microaneurysms, and susceptibility-sensitive hemorrhagic markers (22, 23, 25, 29). AI studies have shown that MMD-related imaging patterns are learnable and that clinical and imaging features can be combined for exploratory risk classification (36–43, 45–47, 49–51). However, no study has yet demonstrated that a model using 7 T-derived biomarkers improves prospective prediction, treatment selection, or patient outcomes. At present, 7 T MRI provides candidate disease-specific imaging features, while AI offers a potential strategy for their quantitative analysis and integration; the combined workflow remains clinically unvalidated.

The maturity of the evidence differs across clinical domains. Hemorrhagic phenotyping has the strongest disease-specific 7 T foundation because deep collateral abnormalities, ventricular microaneurysms, and microbleeds are linked to recognized mechanisms of bleeding (10–12, 22, 23, 25). Evidence for ischemic-risk prediction is less direct because hemodynamic failure is influenced by transit delay, cerebrovascular reserve, collateral compensation, blood pressure, and treatment. Cognitive applications remain further from clinical validation because candidate imaging markers have not been consistently linked to standardized longitudinal neuropsychological outcomes.

### 6.2 Better visualization versus clinical utility

Whether the additional abnormalities detected at 7 T improve clinical decision-making remains uncertain. While 7 T imaging excels at revealing microvascular details missed at 3 T, this increased sensitivity brings clinical dilemmas. Detecting subtle abnormalities of uncertain significance may prompt repeat angiography, intensified surveillance, or treatment escalation without established prognostic benefit. Improved conspicuity is therefore an imaging endpoint, not a patient-centered endpoint.

Future studies should determine whether 7 T-derived markers improve prediction beyond optimized 3 T MRI, DSA, clinical presentation, and hemodynamic assessment. They should also measure whether the information changes decisions regarding angiography, surveillance, or revascularization and whether those changes improve outcomes. Until such evidence is available, 7 T MRI is best considered an adjunctive tool for selected questions rather than a replacement for routine 3 T MRI or DSA (14, 17, 18, 26, 27).

Recent lower-field reconstruction research adds another dimension to this controversy. Omori et al. reported that deep-learning reconstruction improved visualization of moyamoya vessels on 1.5 T and 3 T MRA, with reconstructed 1.5 T images receiving higher quality rankings than original 3 T images in a small retrospective cohort (58). This does not show that lower-field reconstruction reproduces the biological information available at 7 T, and reconstruction-related changes in angiographic scoring require careful validation. It does show that field strength is not the only route to better vessel depiction. For many centers, AI-enhanced acquisition or reconstruction at 1.5 T or 3 T may be more scalable than universal access to 7 T, creating a legitimate competing development pathway.

### 6.3 From diagnostic AI to clinical decision support

Most published AI studies have addressed MMD detection, often in comparisons with healthy controls. These studies demonstrate that disease-related imaging patterns are learnable, but their incremental value in specialist neurovascular practice is uncertain. More clinically relevant tasks include differential diagnosis, collateral quantification, prediction of future events, and assessment after revascularization.

Published performance estimates illustrate both the promise and the limitations of the current evidence. Kim et al. reported an internal-test accuracy of 84.1%, sensitivity and specificity of 0.84, and an AUROC of 0.91 for skull-radiograph-based MMD detection, but accuracy decreased to 75.9% in an independent external dataset (36). Lei et al. reported 97.64% accuracy, 96.55% sensitivity, 98.29% specificity, and an AUC of 0.990 for DSA-based recognition of unilateral moyamoya vasculopathy; their multimodal model classified hemorrhagic phenotype with 90.69% accuracy, 94.12% sensitivity, and 89.86% specificity (38). However, these data were retrospective and did not represent prediction of a prespecified future hemorrhagic event. More recently, Lu et al. reported a macro-average AUC of 0.977 on internal MRA test data but 0.880 on external validation, illustrating the expected performance reduction when models are transferred beyond the development cohort (41).

At the aggregate level, a 2026 meta-analysis of seven retrospective cohorts comprising 4,795 participants reported a pooled sensitivity of 0.65 (95% CI, 0.50–0.79), specificity of 0.85 (95% CI, 0.82–0.89), and summary AUC of 0.85 (48). These results highlight why high accuracy or AUC in selected datasets should not be equated with clinical readiness, particularly when sensitivity remains insufficient for reliably identifying high-risk patients.

Risk-prediction models require longitudinal cohorts with a defined baseline, documented treatment, prespecified outcomes, and adequate follow-up. Their performance must be compared with current clinical and imaging assessment, including calibration and decision impact.

A further distinction is needed between prognosis and treatment benefit. A model may accurately predict that a patient has high event risk under observed care without identifying whether that risk will be reduced by revascularization. Retrospective models may reproduce existing treatment-selection patterns because surgery is influenced by symptoms, anatomy, hemodynamics, and center practice. Models intended to guide revascularization must therefore distinguish prediction of natural-history risk from prediction of postoperative outcome or treatment benefit (11, 12, 56).

### 6.4 Accessibility, evidence gaps, and future research

Limited access to 7 T MRI is not merely a logistical issue but a major determinant of the current evidence base. MMD-specific 7 T studies remain concentrated in a small number of expert centers and generally involve modest sample sizes. Differences in scanner platform, coil configuration, spatial resolution, sequence parameters, acquisition coverage, reconstruction, and artifact correction may generate site-specific imaging features that are unrelated to disease biology but can nevertheless be learned by AI models. These factors constrain reproducibility, external validity, and the development of generalizable quantitative biomarkers.

Accordingly, the near-term clinical role of 7 T MRI is more likely to be selective than universal—for example, in cases with unresolved deep collateral anatomy, suspected submillimeter vascular abnormalities, or research-grade microvascular phenotyping after optimized 3 T MRI and DSA. Multicenter studies should prioritize harmonized acquisition and reconstruction, paired 3 T/7 T comparisons, test–retest reliability, interreader agreement, and cross-site external validation. AI-enhanced acquisition or reconstruction at 1.5 T or 3 T may represent a more scalable alternative for centers without 7 T access, but these approaches should be directly compared with 7 T rather than assumed to provide equivalent biological information.

Reference standards and clinical endpoints also require clearer definition. DSA provides detailed information on luminal stenosis and collateral architecture, but it does not directly establish future ischemic, hemorrhagic, or cognitive risk. Expert labeling of small collateral channels and microaneurysm candidates may also vary between readers. Studies should report annotation procedures, reader agreement, and adjudication methods, and should relate imaging findings to prespecified longitudinal outcomes. Ischemic recurrence, intracranial hemorrhage, cognitive decline, postoperative complications, and bypass response represent distinct clinical domains and should not be combined into a single nonspecific severity endpoint.

Treatment, missing data, and population differences further complicate model development. Revascularization modifies hemodynamics and subsequent event risk, while the decision to operate is influenced by symptoms, anatomy, cerebrovascular reserve, and institutional practice. Models that do not account for treatment timing may therefore reproduce existing treatment-selection patterns rather than estimate natural-history risk or treatment benefit. In addition, DSA, perfusion imaging, neuropsychological testing, and genetic data are not obtained uniformly, and complete-case analysis may select an unrepresentative subgroup. External validation should include different scanners, institutions, age groups, disease stages, ancestral backgrounds, and clinically relevant alternative arteriopathies.

Evidence development can proceed in stages. Initial studies should establish the reproducibility of candidate 7 T biomarkers, followed by external validation of prediction models using contemporary reporting standards such as CLAIM and TRIPOD+AI (55, 56). Models intended for clinical use then require prospective evaluation of workflow, safety, and decision impact, as outlined by DECIDE-AI (57). The 2026 meta-analysis reported encouraging overall discrimination for AI-based stroke-event estimation, but all eligible cohorts were retrospective and pooled sensitivity remained limited (48). In the near term, 7 T MRI is more likely to serve as a selective escalation examination for cases that remain unresolved after optimized 3 T MRI or DSA. AI-enhanced acquisition and reconstruction at 1.5 T or 3 T may offer a more scalable alternative in centers without 7 T access, although these approaches should be compared directly rather than assumed to provide equivalent information (58).

### 6.5 Limitations

This article is a narrative review rather than a systematic review. Although a targeted search was performed, study selection may be incomplete, and no formal risk-of-bias assessment or quantitative synthesis was conducted. The evidence base is also heterogeneous. Some cited studies are MMD-specific, whereas others establish technical feasibility of 7 T vascular, susceptibility, or vessel-wall imaging in related cerebrovascular settings. These studies should not be interpreted as direct evidence of clinical benefit in MMD.

The proposed integration framework is necessarily forward-looking because few studies directly combine 7 T-derived MMD biomarkers with AI and longitudinal outcomes. The framework identifies plausible inputs, outputs, and validation requirements but does not represent a clinically validated workflow. Rapid evolution of imaging hardware and AI methods may also change technical feasibility and reporting standards.

## 7 Conclusion

MMD is a heterogeneous arteriopathy in which ischemic, hemorrhagic, and cognitive risks arise from interacting vascular and parenchymal processes. Current evidence supports a selective role for 7 T MRI in characterizing deep collateral remodeling and susceptibility-sensitive microhemorrhagic markers, whereas its hemodynamic, vessel-wall, and microstructural applications remain exploratory. AI may help quantify these features and integrate them with clinical and physiological information, but this approach has not yet been validated as a clinical decision-support pathway.

The clinical value of future AI-assisted analysis of 7 T-derived biomarkers will depend on whether it improves prediction or management beyond optimized 3 T MRI, DSA, and conventional clinical assessment. Multicenter harmonization, longitudinal validation, and prospective evaluation of decision impact are required before such models can enter routine MMD care. AI-enhanced acquisition and reconstruction at lower field strengths may provide a more accessible alternative in centers without 7 T MRI and should be evaluated alongside, rather than assumed to replicate, ultra-high-field imaging.
